# Extra Uterine Pregnancy

**Published:** 1889-06

**Authors:** 


EXTRA-UTERINE PREGNANCY.—1. Its pathology, by Franklin Townsend, M. D. 2. Its diagnosis, by Joseph Price, M. D. 3. Its treatment, by E. E. Montgomery, M. D. 4. Observations—clinical, pthological and surgical, by W. H. Wathen, M. D. 5. A critique of its management, by J. M. Baldy, M. D. 6. The technique of the operation, by John B. Deaver, M. D. 7. Its management when the fetus survives tubal rupture and goes on to the period of viability, by L. S. McMurtry, M. D. 8. Its treatment (concluded), by A. Van der Veer, M. D.

A Discussion. From the Transactions of the American Association of Obstetricians and Gynecologists, 1888. Together with an editorial review of Tait's Ectopic Pregnancy and Pelvic Hematocele, from the Buffalo Medical and Surgical Journal. Philadelphia: Wm. J. Dornal, 1889. Price, 75 cents.

